# Detection of dynamic substrate binding using MRI

**DOI:** 10.1038/s41598-017-10545-1

**Published:** 2017-08-31

**Authors:** Nirbhay N. Yadav, Xing Yang, Yuguo Li, Wenbo Li, Guanshu Liu, Peter C. M. van Zijl

**Affiliations:** 1 Russell H. Morgan Department of Radiology and Radiological Science, The Johns Hopkins University School of Medicine, Baltimore, MD USA; 2 F. M. Kirby Research Center for Functional Brain Imaging, Kennedy Krieger Institute, Baltimore, MD USA; 3 Present Address: Department of Nuclear Medicine, Peking University First Hospital, Beijing, P.R. China

## Abstract

Magnetic Resonance Imaging (MRI) is rarely used for molecular binding studies and never without synthetic metallic labels. We designed an MRI approach that can specifically detect the binding of natural substrates (i.e. no chemical labels). To accomplish such detection of substrate-target interaction only, we exploit (i) the narrow resonance of aliphatic protons in free substrate for selective radio-frequency (RF) labeling and, (ii) the process of immobilisation upon binding to a solid-like target for fast magnetic transfer of this label over protons in the target backbone. This cascade of events is ultimately detected with MRI using magnetic interaction between target and water protons. We prove this principle using caffeine as a substrate *in vitro* and then apply it *in vivo* in the mouse brain. The combined effects of continuous labeling (label pumping), dynamic reversible binding, and water detection was found to enhance the detection sensitivity by about two to three orders of magnitude.

## Introduction

The ability to noninvasively measure drug-target interactions for the evaluation of biological activity and treatment efficacy in individual patients is expected to be a crucial feature of personalized medicine. Currently, the most sensitive modality for imaging substrate-target interactions in patients is nuclear medicine, using tracer concentrations of radiolabeled probes that have sufficient affinity and selectively for a target of interest. Unfortunately, most hospitals do not have such equipment. MRI is a commonly available technology, but, due to its poor detection sensitivity for low-concentration molecules, limited MRI (water-detection) methods are available to study the effects of substrate binding. One approach available for human studies is so-called physiological MRI, where the effect of neuro-receptor stimuli is measured indirectly *via* their hemodynamic consequences^1^, i.e. without molecular specificity. Other approaches have remained limited to animal studies, for instance use of receptor substrates labeled with metallic compounds that increase water relaxation^2, 3^. To our best knowledge, there currently are no molecular MRI approaches that can study the target binding of non-chemically labeled substrates.

Magnetic resonance spectroscopy (MRS) is a powerful tool for studying molecular structure and function. *In vitro* MRS methods are important for small molecule screening and lead optimization in drug discovery. Advantages include applicability to a broad range of proteins and drugs, no requirement for isotopic labelling, and the possibility of directly identifying the binding component from a mixture of ligands^4, 5^. The sensitivity of MRS to different binding mechanisms has allowed the *in vitro* study of ligand binding to receptors via changes in rotational or translational motion^6^ and of receptor recognition via magnetic coupling between ligand and receptor molecules^7^. Of these, magnetization transfer (MT)^8^ is the most commonly used for drug discovery and screening *in vitro* ^5, 9^ and even to study molecular reaction specifics *in vivo* ^10–12^. MT methods rely on selective radiofrequency (RF) irradiation of a nuclear spin pool (generally protons) on one molecule and measuring the transfer of this irradiation to another^13^. RF irradiation either reduces the magnetization of a proton pool (a process called saturation) or excites it, both referred to as magnetic labeling. For example, in saturation transfer difference (STD) experiments^7^, protons on a protein or other macromolecule with a long rotational correlation time (*τ*_c_) are selectively saturated using low power RF pulses. Efficient dipolar transfer (spin diffusion) ensures the immediate spread of this label throughout the macromolecule and its transfer to bound ligand. Chemical exchange between bound and free ligand results in specific MRS detection of the label in free ligand. For a large excess of ligand (approx. 100-1000:1) and sufficiently fast dissociation rates (K_d_ ~ mM-μM range), appreciable increases in sensitivity can be obtained since irradiated bound ligands are continuously being replaced by unlabeled free ligands, resulting in a cumulative buildup of labeled molecules in the solvent. This allows low concentrations (nM – pM) of receptors to be used^5^. While well suited for *in vitro* spectroscopy, the STD sensitivity enhancement is still not sufficient for *in vivo* imaging, because ligands would at most be in the millimolar range to ascertain biocompatibility and avoid toxicity.

**Figure 1:**
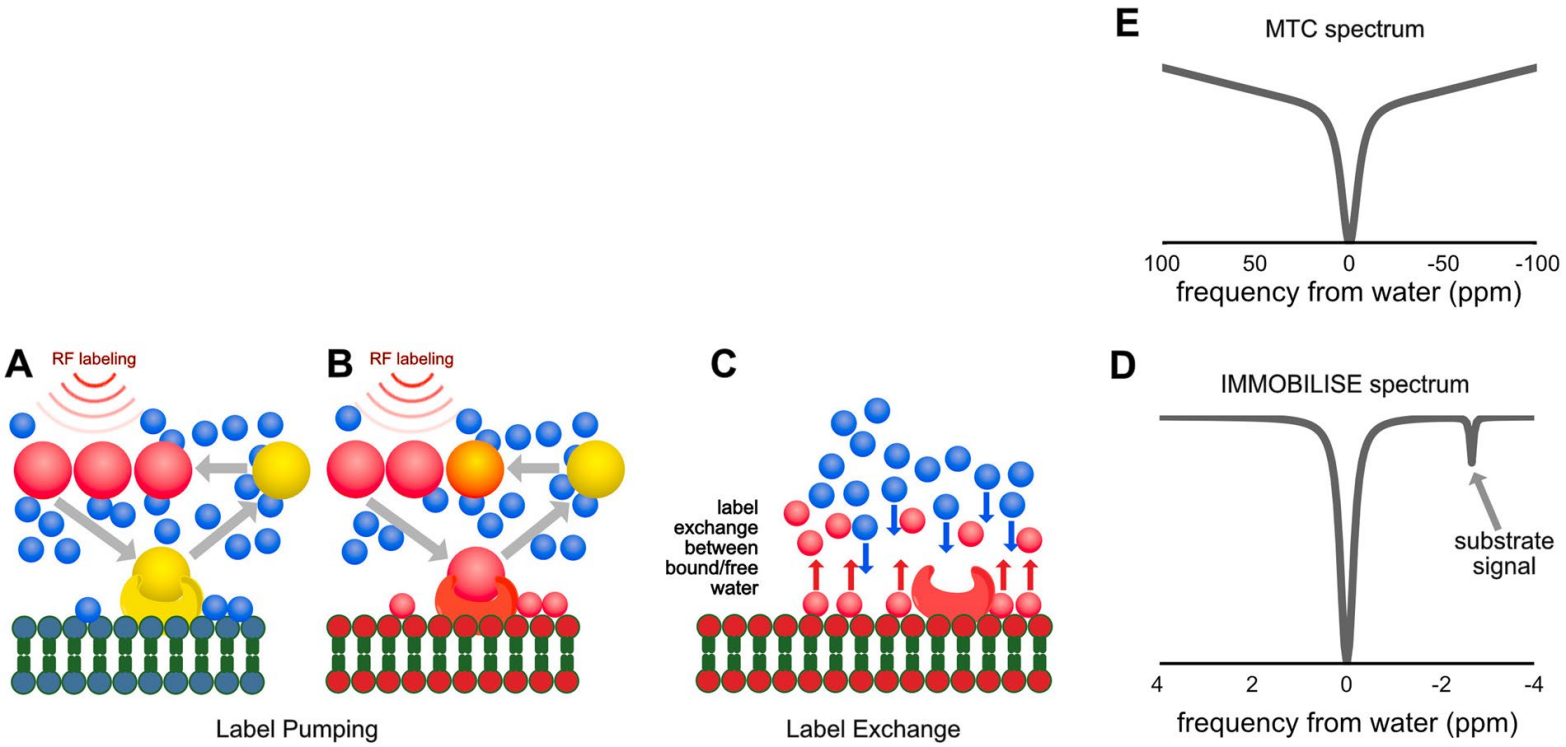
The IMMOBILISE (IMaging of MOlecular BInding using Ligand Immobilization and Saturation Exchange) method. (**A**) Non-exchanging protons (CH, CH2 or CH3) in a low molecular weight molecule (large ball) that exchanges rapidly between free in solution and bound to an immobile target, are labelled in the free state using RF irradiation (indicated in red). (**B**) During binding, the label is rapidly transferred to target protons, after which the substrate is released. Due to continuous irradiation of substrate protons and availability of a large substrate pool, the released molecule is replaced by labeled substrate and the process repeats itself. Spin diffusion rapidly distributes the label over the target protons. (**C**) Ultimately, this label is transferred to the water, either *via* exchangeable protons or through dipolar coupling to protons in bound water molecules, where it appears as signal loss (saturation). Since the water pool is large, the saturated protons or water molecules are replaced by nonsaturated ones and the principle repeats itself. (**D**) The change in water magnetization is detected at the resonance frequency of the substrate. (**E**) This high specificity is not available in conventional magnetization transfer contrast (MTC) experiments that tend to use indiscriminate labeling and strong RF fields.

All of the above MR approaches to detect binding are based on detection of mM signal levels using spectroscopy and, as such, not practical for fast use in the clinic. Early MT studies^14–18^ have shown the existence of a coupling between small metabolites and water that is mediated through nearby semi-solid components. Here we exploit this coupling to design a molecular pump based sensitivity enhancement method that allows the water-based imaging of dynamic binding of actual biological substrates (no chemical modifications) to a macro-molecular target. The principle of the approach, outlined in Fig. 1, is based on the continuous pumping of a magnetic label from substrate to the water detected in MRI that is accomplished through reversible substrate binding to a rigid target molecule (Fig. 1A,B). After efficient transfer through the target, this label ultimately shows up as signal loss (saturation) on the water signal (Fig. 1C). Based on this mechanism, we call this the “IMMOBILISE” approach, for “IMaging of MOlecular BInding using Ligand Immobilization and Saturation Exchange”. We show that we can achieve specificity not only to the substrate of interest but also to the binding process, while retaining the capability to detect the signal with MRI. This method enhances the detection sensitivity with two to three orders of magnitude beyond current *in vitro* binding methods (e.g., saturation transfer difference, STD^7^).

## Results and Discussion

**Figure 2:**
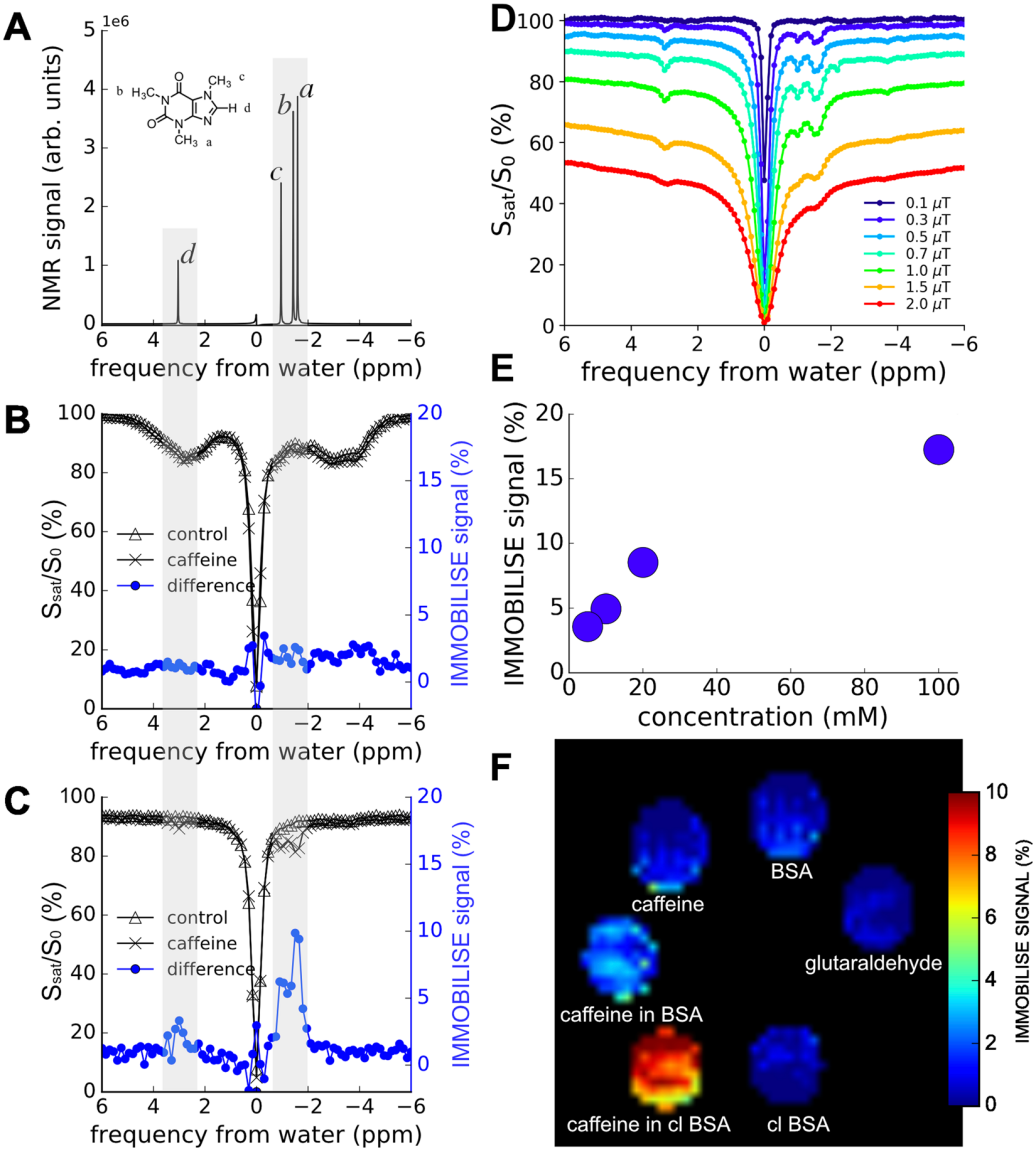
*In vitro* demonstration of the IMMOBILISE approach. (**A**) NMR spectrum of 100 mM caffeine solution. (**B**,**C**) Z-spectra of BSA solution (20% w/w) without (Δ) and with (×) 100 mM caffeine for free (**B**) and cross-linked (**C**) BSA. The difference spectrum (“IMMOBILISE” signal) is shown in blue circles. (**D**) Power dependence of IMMOBILISE Z-spectra using a 3 second saturation pulse. The data show that close to maximum saturation is achieved around 0.5 uT, with loss of the IMMOBILISE signal at much higher B_1_ due to interference from other saturation contributions. (**E**) concentration dependence of caffeine in cross-linked BSA. Above 10% the signal change becomes nonlinear. (**F**) IMMOBILISE-MRI difference maps of 100 mM caffeine in PBS, BSA, and crosslinked BSA and reference tubes of BSA, crosslinked BSA, and glutaraldehyde solution. Maps were generated by integrating IMMOBILISE signal from −1 to −2 ppm. Signal heterogeneity across each tube may be due to low SNR or to the presence of small bubbles present in the BSA mixtures. The frequency in A–D is referenced to the water resonance frequency at 0 ppm (4.7 ppm in NMR spectroscopy).

In Fig. 2 the IMMOBILISE method is applied to caffeine in protein solution, showing that MRI contrast is generated only when there is dynamic binding of RF-labeled caffeine to a rigid target, needed to accomplish efficient transfer of label to bulk water pool. The NMR spectrum of 100 mM caffeine (Fig. 2A) displays peaks of aliphatic protons, including three methyl groups. Figure 2B show Z-spectra (displaying the relative reduction of water intensity as a function of irradiation frequency relative to the water resonance at 0ppm) for a solution of bovine serum albumin (BSA) without and with caffeine. The difference spectrum shows negligible signal at the caffeine frequencies. Strikingly, when the protein is crosslinked using glutaraldehyde, signals corresponding to the caffeine resonances become visible in the water saturation difference spectrum (Fig. 2C), with a linewidth corresponding to mobility in free solution. Figure 2E displays the IMMOBILISE signal in crosslinked BSA at -1.6 ppm as a function of caffeine concentration. The data from this set of phantoms, which were prepared in a different batch from the other data in Fig. 2, show approximately 4% signal for 5 mM up to 17% of the water signal (110 M protons) for 100 mM caffeine. This translates to sensitivity enhancement of between 900x at low concentrations and about 200x at high concentration. This increase in sensitivity is sufficient to generate MR images (Fig. 2F). These maps, showing IMMOBILISE images from caffeine solution (caffeine), BSA solution, caffeine in BSA, caffeine in crosslinked BSA (caffeine in cl BSA), crosslinked (cl) BSA, and glutaraldehyde solution confirm that only caffeine in crosslinked BSA shows high IMMOBILISE MRI contrast. Thus, contrast from caffeine occurs only when the molecule is transiently immobilised, allowing the RF label of caffeine protons to be transferred to water not directly, but via the semisolid matrix. The signal cannot be from caffeine bound to the semisolid, as the NMR linewidth would be so broad to make the signals invisible. The signal sensitivity is enhanced because the transfer is continuously refreshed through a label pumping mechanism that exploits the reversible binding. This opens up the exciting possibility to study the binding kinetics of substrates to solid-like structures (e.g. membranes or receptors on there).

**Figure 3:**
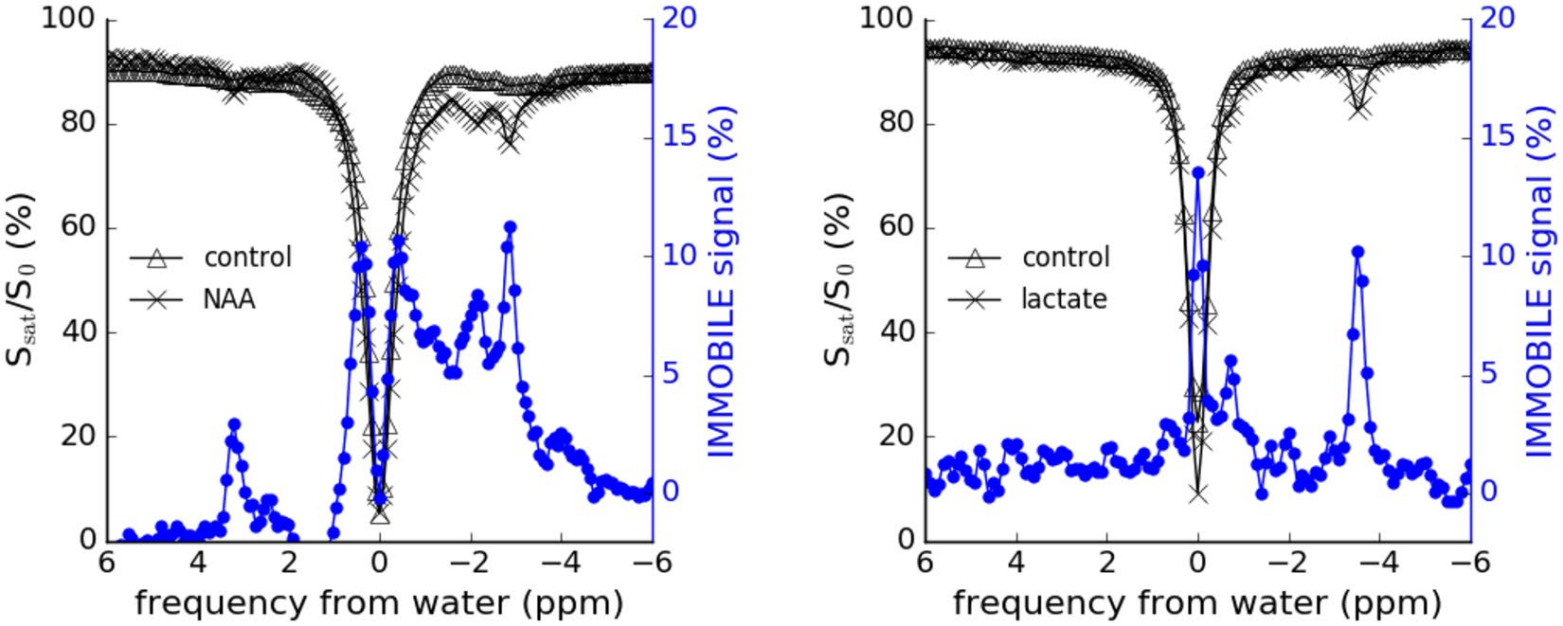
IMMOBILISE data from 100 mM N-acetylaspartate (NAA) and 100 mM lactate in crosslinked BSA phantoms. Notice the high-resolution IMMOBILISE-based differences agreeing with the spectral appearance for these compounds known from MRS, namely the NAA CH_3_ resonance at −2.7 ppm, CH_2_ resonances between −2.2 to −2.0 ppm and the NH resonance at 3.2 ppm (which is temperature dependent), corresponding to the known MRS frequencies at 2.0 ppm, 2.5–2.7 ppm and 7.9 ppm. For lactate, the CH_3_ and CH peaks at −3.4 ppm and −0.6 ppm (MRS: 1.3 and 4.1 ppm) respectively are visible.

To further illustrate the method, we show IMMOBILISE data for 100 mM N-acetylaspartate (NAA) and 100 mM lactate in crosslinked BSA (Fig. 3). Here, several non-exchangable peaks commonly measured in MRS are apparent. In the Z-spectra, the aliphatic protons of NAA are observed between −2.7 to −2.0 ppm and the NH resonance at 3.2 ppm from water, corresponding to the 2.0 to 2.7 ppm range and 7.9 ppm resonance in MRS. Note that this NH frequency is temperature dependent. Also clearly apparent are the CH3 (−3.4 ppm) and CH (−0.6 ppm) peaks from lactate, corresponding to 1.3 ppm and 4.1 ppm in proton MRS.

The phantom experiments in Figs 2 and 3 show that very low pulse powers (~0.5 μT) are sufficient for generating IMMOBILSE contrast. This makes sense as non-exchangeable protons are irradiated in small molecules where the speed of dipolar transfer to neighboring protons in very small. Thus a large pool of saturated protons is built up in a reservoir that can continuously pump saturation into the binding site. As such, once the saturation time is sufficiently long, there will be a negligible dependence on the B_1_ power needed. This is illustrated in Fig. 2D. In addition to providing a situation in which SAR deposition will not be a concern, using such low pulse powers has the advantageof  high specificity since other types of magnetization transfer contrast are minimized. Thus the difference spectra appear with a spectral resolution very similar to that in MR spectroscopy.

**Figure 4:**
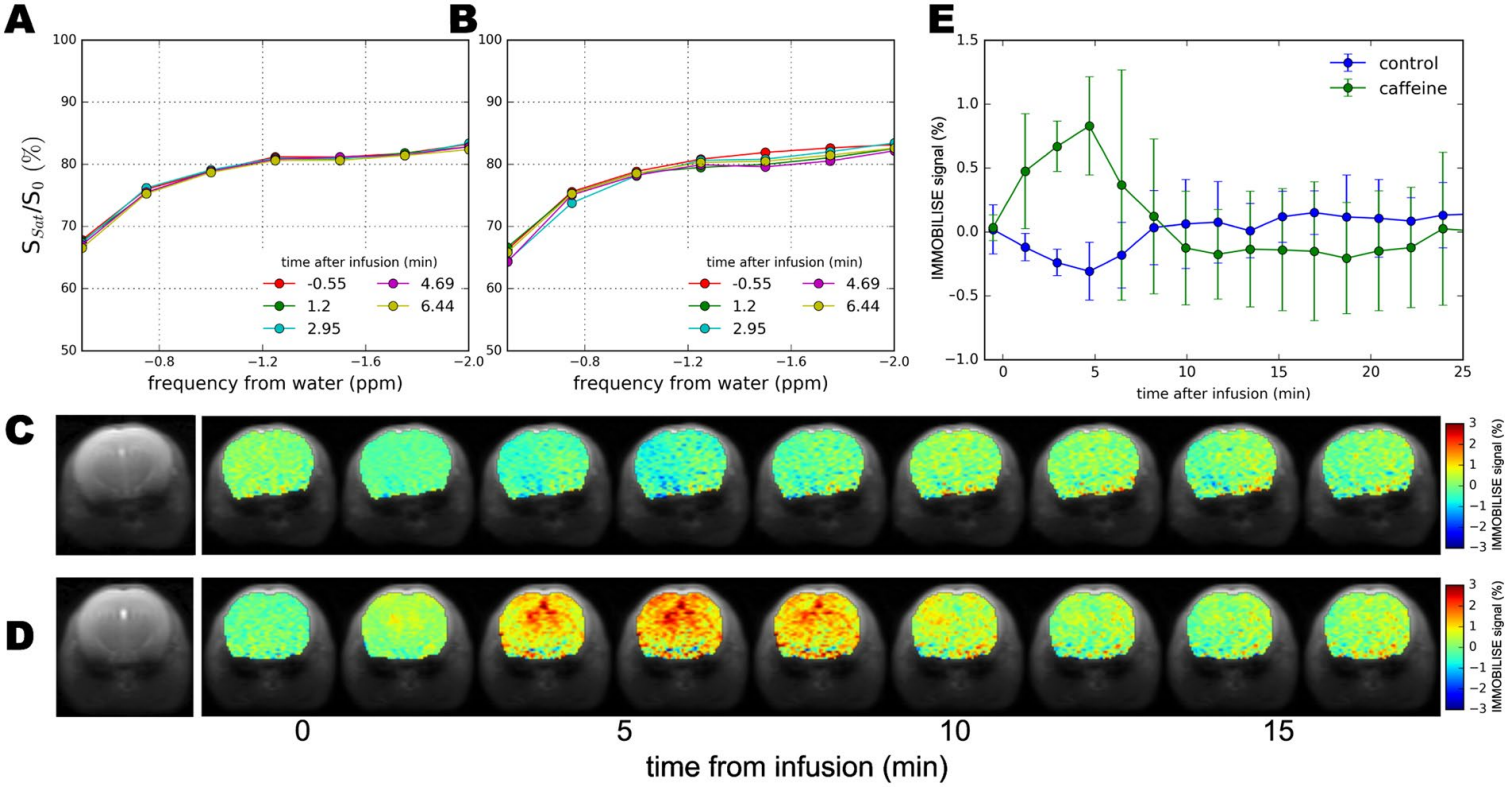
*In vivo* imaging from mice injected with caffeine. (**A**,**B**) Z-spectra of thalamus before and after 20 s infusion of (A) PBS (50 µL) and (**B**) caffeine (50 µL, 100 mM). Time 0 is after infusion stops. (**C**) Dynamic IMMOBILISE images (at the −1.5 ppm frequency) after (**C**) PBS and (**D**) caffeine infusion. (**E**) Mean and standard deviation of dynamic signal differences in mouse thalamus for PBS (n = 3) and caffeine (n = 5). The larger error bars for the caffeine data may be due to motion.

We tested the IMMOBILISE approach *in vivo* in mice using a PBS-solution of caffeine and PBS controls. A small reduction in signal was found for mice injected with just PBS (Fig. 4A,C), in line with recent literature on saline showing such effects on MR signal^19^. In contrast, for mice injected with caffeine, noticeable increases were visible in the thalamus over the caffeine spectral region (Fig. 4B,D). Looking at the average signal change for all mice, the dynamic signal change persisted for about 10 minutes after infusion (Fig. 4C). This time course excludes the possibility of the effect being due to perfusion, which typically shows contrast over much shorter time frames ( < 1 min^20^). Although cerebral blood flow (CBF) and BOLD changes occur after caffeine administration^21^, the IMMOBILISE contrast is obtained from images normalized with respect to the water signal without saturation and thus should be independent of CBF and BOLD changes. Such independence is futher confirmed by the matching within error of the off-resonant Z-spectral data points between −0.8 and −1.2 ppm before and after injection. The concentration of caffeine in mouse blood was 2.3 mM, 5 minutes after the infusion of 50 µL of 100 mM caffeine, with a detection sensitivity of about 1–3% change in water signal. The higher sensitivity enhancement *in vivo* could be due to higher binding site density and different binding kinetics compared to the BSA phantoms. For a sufficient supply of labeled ligand, the amount of enhancement is proportional to the amount of binding-transfer events that take place during the irradiation period. As labelling is transferred rapidly during binding, the sensitivity of this technique, i.e. the efficiency of the pump, is largely determined by the dissociation rate of the molecule. An important task in future studies will be the development of suitable reference phantoms for specific and non-specific binding that more closely mimic the *in vivo* environment. The BSA phantoms used here are not meant to replicate *in vivo* conditions and even may be difficult to replicate exactly between batches.

The IMMOBILISE approach exploits some principles inherent in chemical exchange saturation transfer (CEST) imaging, namely pool replenishment through exchange^22, 23^, but is inherently very different as the contrast enhancement occurs in two steps, one of which does not involve exchangeable protons but molecular binding. In addition, after an initial period, there is a continuous pool of saturated substrate available, while in CEST the non-saturated proton that has replaced the exchange saturated proton has to be again saturated. While we do not yet have a quantitative model describing the IMMOBILISE signal, we anticipate from the results that this technique is ideally suited for imaging low-affinity binding of small to intermediate sized molecules (relatively long T2 and thus narrow spectral peaks when unbound). In addition, the IMMOBILISE method can isolate the signal from substrates with high specificity, since the resonance frequency is observed at the substrate-specific proton frequency in the Z-spectrum (Fig. 1D) and low power is used. While this seems to be contradicted by the result of an increased signal throughout the brain in the caffeine distribution maps, this finding is in line with expectation, because caffeine passes freely through most biological membranes, including the blood-brain barrier^24^. There are however brain regions with slightly higher signal. These regions include the thalamus and cortex, which have elevated levels of the adenosine receptor A_1_ that caffeine is known to bind to^25, 26^.

It is important to mention that small molecules *in vivo* will experience similar “IMMOBILISE” effects. As mentioned above, early MT studies^14–18^ have noticed the coupling between small molecules and water mediated through the semi-solid component. This phenomenon therefore will contribute to both the aliphatic and aromatic regions of endogenous Z-spectra including even the signals of very slowly exchanging amide protons, such as the one from NAA (See Fig. 3). The aliphatic region therefore has multiple contributions, some of which are slowly being identified^27^ and the origin of which is slowly becoming clearer^28^. While these resonances may complicate the interpretation of CEST MRI experiments, particularly when MTR asymmetry analysis^29^ is used to quantify CEST contrast, the additional information can most likely be used for new applications in the future. Further, since very low pulse powers are used for this molecular labelling, we presume this technique should easily be translatable to human field strengths.

Many if not most of the processes involved in biological activity involve formation of two-component complexes. We therefore expect the IMMOBILISE method to have important applications in the fields of drug development and testing, enzyme binding, theranostics, and the study of receptor substrate binding. Only few methods exist for imaging drugs that are relatively weakly bound. This is an important category of drugs since drugs with faster dissociation rates can increase the therapeutic index. Faster binding drugs may target short-lived receptors more effectively^30^. Due to its dynamic character, the IMMOBILISE technique is complementary to nuclear imaging studies where imaging often takes place at a certain time period (up to several hours) after radiotracer injection before imaging.

## Methods

### *In vitro* Experiments

*In vitro* experiments to demonstrate specificity of substrate binding to a rigid lattice were performed on caffeine (100 mM), N-acetylaspartate (100 mM), and lactate (100 mM) solutions mixed with crosslinked 20% (w/w) bovine serum albumin (BSA). BSA crosslinking was done using 25 μL/mL glutaraldehyde solution^15^. Separate batches of caffeine phantoms in crosslinked BSA were prepared at concentrations of 5, 10, 20, and 100 mM. Each solution was prepared at pH 7.4. MRI scans on these phantoms were done on a 17.6 T Bruker Avance III (Bruker Biospin, Ettlingen, Germany) at 37°C using a 3 s CW saturation pulse followed by a single shot fast spin echo (FSE) MRI readout. The echo time (TE) and repetition time (TR) were set to 4 ms and 6 s respectively. The irradiation frequency was stepped over the proton spectral range (±6 ppm in steps of 0.15 ppm) and a so-called Z-spectrum^29^ of the induced relative water saturation as a function of irradiation offset was detected. When using imaging, each voxel will provide such a Z-spectrum.

### *In vivo* Experiments

All experiments were performed in accordance with Institutional Animal Care and Use Committee guidelines and approved by the Johns Hopkins University Animal Care and Use Committee. Eight 6-8 week old female BALB/c mice, were anesthetized by isoflurane and kept warm with a heating pad and kept in place with a stereotactic holder. The tail vein was cannulated for administration of a caffeine solution (100 mM in PBS with pH 7.4). A home-built catheter was connected to a syringe infusion through PE-50 tubing. Two groups of mice were infused with either 50 μL of caffeine solution (*n* = 5) or PBS (*n* = 3) over a 20 s duration with a dose of 0.2 mmol/kg.

MRI experiments were acquired on a 11.7 T Bruker Biospec preclinical scanner (Bruker, Ettlingen, Germany) equipped with a 72 mm quadrature volume resonator for RF transmission and a 2 × 2 mouse phased array coil for RF reception. The saturation preparation consisted of a 3 s, 0.5 μT CW saturation pulse followed by a two-shot FSE readout. The FSE readout parameters were TR/TE = 6000/4 ms, a 64×64 acquisition matrix across a field of view of 17×17 mm and slice thickness of 1 mm. Prior to a bolus infusion of either phosphate buffered saline (PBS) or caffeine, a complete Z-spectrum was acquired with saturation frequencies between ±4 ppm and a partial spectrum with frequencies at −0.50, −0.75, −1.00, −1.25, −1.50, −2.00. Acquisition of each partial Z-spectra took 105 s and was continued for 1-hour post bolus and then a complete Z-spectrum was again acquired. Blood caffeine concentration was measured in a mouse at 5 minutes post-infusion using the method described by Alkaysi *et al*.^31^.

### Analysis of data

In order to study the possibility to specifically detect binding, we studied the difference between MRI images and Z-spectra of phantoms with and without caffeine *in vitro* and before and after infusion of a caffeine solution *in vivo*. *In vivo* data was analyzed by subtracting the signal after caffeine infusion from the reference scans acquired prior to infusion. The *in vivo* data at each time point was normalized by a reference image acquired at that time point. Maps were generated by mapping the signal difference (pre minus post infusion) between the partial spectra (−1.0 to −2.0 ppm). Field correction for static field (*B*_0_) inhomogeneities was done by creating field maps from the low power Z-spectra acquired in our study and shifting the Z-spectra voxel wise according to the WASSR method^32^.

### Data availability

The datasets generated during and/or analysed during the current study are available from the corresponding author upon request.
